# FE Analyses of Hyperelastic Solids under Large Bending: The Role of the Searle Parameter and Eulerian Slenderness

**DOI:** 10.3390/ma13071597

**Published:** 2020-04-01

**Authors:** Federico Oyedeji Falope, Luca Lanzoni, Angelo Marcello Tarantino

**Affiliations:** 1 Department of Engineering Enzo Ferrari, DIEF, University of Modena and Reggio Emilia, via P. Vivarelli 10, 41125 Modena, Italy; federicooyedeji.falope@unimore.it (F.O.F.); luca.lanzoni@unimore.it (L.L.); 2 Centro di Ricerca Interdipartimentale Costruzioni e del Territorio, CRICT, via P. Vivarelli 10, 41125 Modena, Italy

**Keywords:** Finite elements, finite bending, 3D elasticity, Eulerian slenderness, compactness index, Searle parameter, Elastica

## Abstract

A theoretical model concerning the finite bending of a prismatic hyperelastic solid has been recently proposed. Such a model provides the 3D kinematics and the stress field, taking into account the anticlastic effects arising in the transverse cross sections also. That model has been used later to extend the Elastica in the framework of finite elasticity. In the present work, Finite Element (FE) analyses of some basic structural systems subjected to finite bending have been carried out and the results have been compared with those provided by the theoretical model performed previously. In the theoretical formulation, the governing equation is the nonlinear local relationship between the bending moment and the curvature of the longitudinal axis of the bent beam. Such a relation has been provided in dimensionless form as a function of the Mooney–Rivlin constitutive constants and two kinematic dimensionless parameters termed Eulerian slenderness and compactness index of the cross section. Such parameters take relevance as they are involved in the well-known Searle parameter for bent solids. Two significant study cases have been investigated in detail. The results point out that the theoretical model leads to reliable results provided that the Eulerian slenderness and the compactness index of the cross sections do not exceed fixed threshold values.

## 1. Introduction

The nonlinear bending theory of elastic bodies has attracted a lot of interest because of its relevance in many physical and engineering applications. As an example, in the past decades, the diffusion of robotic technologies has demanded the precise knowledge of the mechanical response of technological components subjected to large bending.

The soft robots [1], i.e., robots based on extremely compliant components, are used to produce pneumatic robots [2], to simulate artificial systems [3], animals [4], human hands [5] and other gripper devices [6]. For these kinds of high-tech applications, the mechanical role of the external load is played by the light [7], humidity [8] or electricity [9] to drive motion.

In the aforementioned and many other applications, the theory of elastic bending allows predicting strains and stresses in the deformed solid. For situations in which the displacement and strains are small, the classical linearized elasticity theory provides reliable results. In such a framework, a certain number of closed-form solutions are available for beams [10], shells and plates [11,12] under different loading and boundary conditions. A few closed-form solutions can be found also for the 3D elastic bodies, with special reference to symmetric layouts [13,14].

However, linear elasticity cannot be used to properly assess the mechanical response of bodies that exhibit large displacements and/or strains, like, for example, tentacle action or hand closure in soft robots. For these and other contexts the finite elasticity is much more appropriate than the linearized theory.

From a mathematical standpoint, a large number of challenging insights are involved in the finite theory. Nevertheless, also in the framework of nonlinear elasticity, a certain number of analytical solutions in the context of homogeneous deformations are available for some basic layouts, like prismatic bodies under axial dead loads [15,16,17,18] or shear loads [19].

Concerning finite bending, various studies can be found in Literature [20,21,22]. Such studies are typically approached through the semi-inverse method: Some geometrical assumptions about the kinematics allows obtaining the displacement field, which is definitely assessed by solving a boundary value problem provided by the equilibrium conditions. However, all the aforementioned works have been carried out by assuming plane strain or plane stress conditions, thus reducing the problem of the finite bending of solids to a 2D problem.

Recently, the Rivlin formulation [20] has been extended to a 3D framework by taking into account also the anticlastic deformation arising in the transverse cross sections of the solid subjected to uniform bending [23]. Later, the analysis has been extended to beams subjected to variable bending moment [24,25]. In that study, a nonlinear relation between the bending moment and the curvature of the longitudinal axis has been found for a compressible Mooney–Rivlin material. As a matter of fact, this relation represents a generalization of the well known Elastica [26] in the context of finite elasticity.

In the present work, based on the theoretical formulation reported in [23,24], some study cases are analytically investigated. In particular, the applications consist of a clamped beam subjected to a couple or a shear force acting at its free end. The nonlinear relation between the bending moment and the curvature of the longitudinal axis is written in a dimensionless form, thus highlighting the relevance of the Eulerian slenderness together with a second dimensionless parameter, which stands for the compactness of the transverse cross sections. Such parameters are coupled into the well known Searle parameter [27,28]. The theoretical results in terms of displacements of the longitudinal axis, stretch and stress distributions within the cross sections are compared with the results provided by Finite Element (FE) simulations. The comparison allows assessing the reliability of the theoretical model to predict accurately the mechanical response of beams under large bending based on fixed threshold values of the governing parameters.

The paper is organized as follows: In Section 2 a brief remark about the theoretical model [23] is provided, with particular emphasis on the basic assumptions concerning the kinematics. The governing equation in terms of moment-curvature relationship is then provided in dimensionless form based on the definition of the Eulerian slenderness and the compactness index of the cross sections. The main results obtained by the theoretical model and the FE simulations are compared in Section 3 through the investigation of two study cases. In that Section, the role played by the Eulerian slenderness and the compactness of the cross sections is discussed in detail and relevant threshold values of the governing parameters are found. Finally, conclusions are drawn in Section 4.

## 2. The Theoretical Model for the Finite Bending of Solids

### 2.1. Remarks on the Theoretical Model

**Figure 1:**
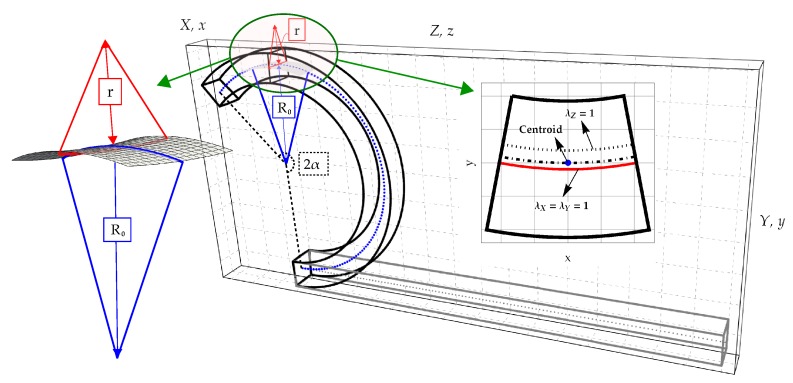
Prismatic hyperelastic solid under bending: Kinematic parameters, anticlastic surface and deformed configuration of the cross section.

In the present Section the basic assumptions of the theoretical model are briefly recalled [23]. Let us consider a prismatic body of length *L*, width *B* and height *H*, placed in a Cartesian reference system {O,X,Y,Z}. The solid is uniformly bent around the *X*-axis by imposing a prescribed rotation angle $2\alpha_{0}=L/R_{0}$ at the end cross sections, as sketched in Figure 1.

Hereinafter $R_{0}$ denotes the radius of curvature of the centroidal line (X=0,Y=0) in the ZY-plane, which is constant along the longitudinal *Z*-axis, see Figure 1. The kinematics is such to distinguish the centroidal line from the longitudinal neutral fibre ($X=0,Y=QM:\;\lambda_{Z}=1)$ and from the transverse neutral plane ($Y=QN:\;\lambda_{X}=\lambda_{Y}=1$, being $\lambda_{J}$ the stretch along the *J*-axis).

The kinematics is based on the following three basic hypotheses [23]:

1. the longitudinal fibres, parallel to *Z*-axis, after bending are deformed into arcs of circumferences (blue curves in Figure 1). As reported above, the longitudinal radius of curvature of the deformed centroidal fibre is denoted as $R_{0}$, whereas the longitudinal fibre with unitary transverse stretches ($\lambda_{X}=\lambda_{Y}=1$) is characterized by the longitudinal radius of curvature *R*;
2. during bending, transverse cross sections belonging to XY planes preserve their planarity and exhibit the same deformation;
3. solid transverse fibres, parallel to *X*-axis, after bending are deformed into arcs of circumferences (red curves in Figure 1). The transversal fibre with unitary transverse stretches ($\lambda_{X}=\lambda_{Y}=1$), is characterized by the anticlastic (or transverse) radius if curvature *r*.

Therefore, conversely to existing models on the 2D finite bending [20,21,22], in the theoretical formulation proposed in [23] a complete 3D description of the kinematics is provided. Due to bending, both the longitudinal and anticlastic curvatures of each fibre of the solid are considered. As a result, all components of the strain and stress fields are provided.

The second hypothesis is widespread in the framework of the linearized beam theory and it is known as Euler–Bernoulli assumption. As shown in [29,30], such an assumption is still valid at large displacements and strains. It is remarked that the proposed formulation neglects the deformation induced by shearing or axial loads.

From the third hypothesis about the anticlastic radius of curvature, it follows the invariance of the transverse stretch $\lambda_{X}$ and $\lambda_{Y}$ with respect to (w.r.t.) the *X*-variable. However, as shown in the following, such an assumption depends on the governing parameters.

Let us define $\beta_{C}=B/H$ as the *compactness index* of the cross sections. As already pointed out by Lamb [28] and experimentally by Searle [27], the cross sections of an elastic bent solid exhibit a variable curvature along the solid width. Such a variation increases in the neighborhood of the corners of the cross section.

The equilibrium condition, as shown in [23], is exactly fulfilled for the centroidal fiber in terms of local equilibrium equations, whilst it is satisfied in average at the boundaries. Therefore, moving away from the centroidal fiber, the equilibrium fulfillment loses its accuracy, but with negligible errors in the case of slender solids with compact cross sections [24].

The concept of slenderness can be referred to the Lagrangian or Eulerian configurations. In the classical beam theory [10], the Lagrangian slenderness is defined as the ratio $\beta_{LS}=L/\max\left(B,\;H\right)$ [10]. Generally, a beam can be considered slender if $\beta_{LS}\geq 10$ regardless the magnitude of strain or displacement fields involved in the deformation process (a definition of *Eulerian slenderness* $\beta_{ES}$ will be provided in Section 2.2 based on the nonlinear moment-curvature relationship, showing that the bending problem is governed by two parameters: $\beta_{ES}$ and $\beta_{C}$).

In the following we consider a hyperelastic prismatic solid characterized by a stored energy density function $\omega_{MR}$, here assumed according to that of a compressible Mooney–Rivlin (MR) material [31]. The stored energy density function of an isotropic material is expressed as a function of the invariants $I_{1},I_{2},I_{3}$ of the right Cauchy–Green strain tensor $C=F^{T}\;F$ as follows (for any details in terms of symbols and notation the Reader is referred to [23]):

(1) $$\omega_{MR}\left(I_{1},I_{2},I_{3}\right)=a\left(I_{1}-3\right)+b\left(I_{2}-3\right)+c\left(I_{3}-1\right)-\left(a+2\;b+c\right)\ln\left(I_{3}\right),$$

with

$$I_{1}=\lambda_{X}^{2}+\lambda_{Y}^{2}+\lambda_{Z}^{2},\;\;I_{2}=\lambda_{X}^{2}\lambda_{Y}^{2}+\lambda_{X}^{2}\lambda_{Z}^{2}+\lambda_{Y}^{2}\lambda_{Z}^{2},\;\;I_{3}=\lambda_{X}^{2}\lambda_{Y}^{2}\lambda_{Z}^{2},$$

where a,b and *c* denote the constitutive MR parameters.

Kinematics, equilibrium conditions and constitutive law allows assessing the displacement field s(X,Y,Z)=ui+vj+wk as [23]

(2) $$s=\left[\begin{array}{c} -X+r\;e^{-\frac{X+QN}{r}}\;\sin\frac{X}{r}\\ -Y-R-QN+\left[R+r\left(1-e^{-\frac{Y+QN}{r}}\cos\frac{X}{r}\right)\right]\;\cos\frac{Z}{R_{0}}\\ -Z+\left[R+r\left(1-e^{-\frac{Y+QN}{r}}\cos\frac{X}{r}\right)\right]\;\sin\frac{Z}{R_{0}} \end{array}\right],$$

which is completely known once the following nonlinear system

(3) $$\left\{\begin{array}{c} r\left[R_{0}^{2}\left(a+2b\right)-aR^{2}+c\left(R^{2}+R_{0}^{2}\right)\right]-2R\left[R_{0}^{2}\left(a+3b\right)+c\left(R^{2}+R_{0}^{2}\right)\right]=0\\ R_{0}-R=r\left(1-\cos\frac{B}{2r}\right)\\ QN=r\log\left(\cosh\frac{H}{2r}\right) \end{array}\right..$$

It is remarked that Equation (3)_1_ follows by imposing the equilibrium condition, $\mathrm{Div}\;T_{R}=0$, along the *Y* and *Z* directions. Equation (3)_2_ is obtained by imposing that the lateral surface of the bent solid is unloaded, i.e., the Piola-Kirchhoff stress vector, $t_{R}=T_{R}n$, must be 0 on the contour of each cross section (n is the outward unit normal). Finally Equation (3)_3_ comes from a simplifying assumption about stretch $\lambda_{Y}$ along *Y* direction (for details, see [23]). Therefore system (3) is solved in the unknown kinematic parameters R,r and QN, and then the principal stretches can be evaluated as the roots of the diagonal components of the right Cauchy-Green strain tensor [23]

(4) $$\lambda_{X}=\lambda_{Y}=e^{-\frac{QN+Y}{r}},\;\lambda_{Z}=\frac{r(1-e^{-\frac{QN+Y}{r}}\cos\frac{X}{r})+R}{R_{0}}.$$

Once the displacement field (2) is known, both the (first) Piola–Kirchhoff $T_{R}$ and Cauchy T stress tensors can be readily obtained as (for details about the expression of the Piola–Kirchhoff stress tensor, see Equations (30) and (56) of [23]. Analogous expressions for the Piola–Kirchhoff stress tensor are given also in [21,31])

(5) $$T_{R}=R\;S,\;T={\left(F^{*}\right)}^{-1}\;T_{R},$$

where symbol ^(*)^ stands for the cofactor and tensors $R^{T}=R^{-1}$ and S turn out to be

$$\left[R\right]=\left[\begin{array}{ccc} \cos\frac{X}{r} & -\sin\frac{X}{r} & 0\\ \cos\frac{Z}{R_{0}}\sin\frac{X}{r} & \cos\frac{X}{r}\cos\frac{Z}{R_{0}} & -\sin\frac{Z}{R_{0}}\\ \sin\frac{X}{r}\sin\frac{Z}{R_{0}} & \cos\frac{X}{r}\sin\frac{Z}{R_{0}} & \cos\frac{Z}{R_{0}} \end{array}\right],\;\left[S\right]=\left[\begin{array}{ccc} S & 0 & 0\\ 0 & S & 0\\ 0 & 0 & S_{Z} \end{array}\right],$$

with

$$\begin{array}{c} S=2e^{-\frac{3\left(QN+Y\right)}{r}}\left[-\left(a+2b+c\right)e^{\frac{4\left(QN+Y\right)}{r}}+ae^{\frac{2\left(QN+Y\right)}{r}}+b\right]+\frac{2e^{-\frac{5\left(QN+Y\right)}{r}}\left[be^{\frac{2\left(QN+Y\right)}{r}}+c\right]{\left[\left(r+R\right)e^{\frac{QN+Y}{r}}-r\cos\frac{X}{r}\right]}^{2}}{R_{0}^{2}},\\ S_{Z}=\frac{2e^{-\frac{5\left(QN+Y\right)}{r}}\left[\left(r+R\right)e^{\frac{QN+Y}{r}}-r\cos\frac{X}{r}\right]}{R_{0}}\left\{ae^{\frac{4\left(QN+Y\right)}{r}}+2be^{\frac{2\left(QN+Y\right)}{r}}+c-\frac{R_{0}^{2}\left(a+2b+c\right)e^{\frac{6\left(QN+Y\right)}{r}}}{{\left[\left(r+R\right)e^{\frac{QN+Y}{r}}-r\cos\frac{X}{r}\right]}^{2}}\right\}. \end{array}$$

Relations (1-5) will be used in Section 3 to compare the FE results with the theoretical predictions in terms of deformed configurations and stretch and stress distributions within the cross section.

### 2.2. Generalization to Variable Bending Moment

**Figure 2:**
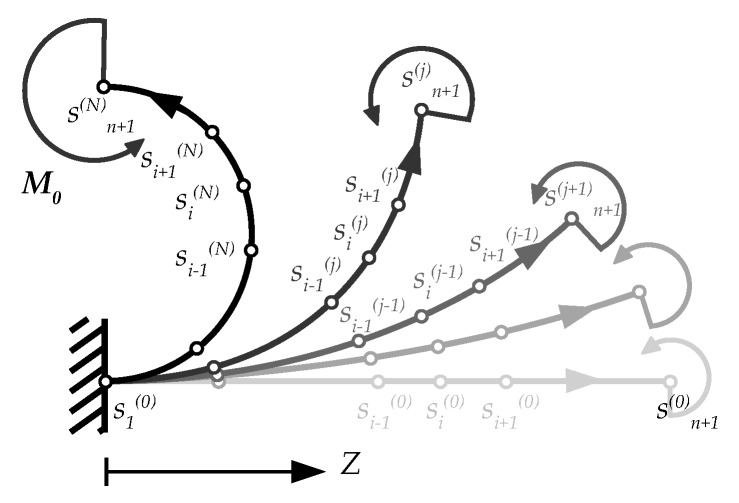
Discretization of the beam axis: Location of nodes at different iterations.

The theoretical model [23] was extended to the cases of nonuniform bending of slender beams in [24] (in [24], the deformed configuration of the centroidal fibre is described by the curvilinear abscissa *s* as sketched in Figure 2. Therefore, both the bending moment and the radii of curvature *R* and *r* turn out to be functions of the deformed beam axis, i.e., $m_{x}=m_{x}\left(s\right)$, $R_{0}=R_{0}\left(s\right)$ and r=r(s). Likewise [24], here $R_{0}$ is referred to the longitudinal neutral fibre corresponding to Y=0, for which $\lambda_{Z}=1$. In other words, the simplifications $R_{0}=R$ and QN=0 assumed in [24] is adopted here. Therefore, in the present work, the longitudinal axis of the beam corresponds to the fibre at X=0,Y=0,Z=Z). In particular, the nonlinear relation between the internal bending moment (moment due to the Eulerian stress $T_{3}$ over the transverse cross section [24]) and the longitudinal radius of curvature $R_{0}$ is given as

(6) $$m_{x}\left(s\right)=\frac{E_{\mathrm{MR}}I_{X}}{R_{0}\left(s\right)}+\frac{10}{3}{\left[\frac{6B^{2}}{R_{0}\left(s\right)H}\right]}^{3}\sum_{i=1}^{2}g_{i}+O\left(R_{0}^{-5}\right),$$

where *s* is the curvilinear coordinate along the deformed configuration of the longitudinal axis, $I_{X}=BH^{3}/12$ is the second moment of inertia of the (undeformed) cross section and

$$g_{i}=\frac{H^{2(i+2)}}{B^{2i+1}}E_{\mathrm{MR},i},$$

are parameters in which $E_{\mathrm{MR},i}$ are defined as follows:

$$\begin{array}{c} E_{\mathrm{MR},1}=5\nu_{\mathrm{MR}}\left\{a\left(6\nu_{\mathrm{MR}}+3\right)+b\left[4\nu_{\mathrm{MR}}\left(5-2\nu_{\mathrm{MR}}\right)+6\right]+c\left[2\nu_{\mathrm{MR}}\left(7-6\nu_{\mathrm{MR}}\right)+3\right]\right\},\\ E_{\mathrm{MR},2}=3\left\{4\nu_{\mathrm{MR}}^{3}\left(8b+23c\right)-\nu_{\mathrm{MR}}^{2}\left(7a+62b+79c\right)-9\nu_{\mathrm{MR}}\left(a+2b+c\right)-6\left(a+2b+c\right)\right\}. \end{array}$$

It is worth nothing that the transition from finite to linearized elastic theory reported in [23] has led to some relations between the MR constitutive parameters and the usual elastic moduli, i.e., the Young modulus $E_{\mathrm{MR}}$ and Poisson ratio $\nu_{MR}$, according to

(7) $$E_{\mathrm{MR}}=\frac{4(a+b)(a+4b+3c)}{a+3b+2c},\;\nu_{\mathrm{MR}}=\frac{b+c}{a+3b+2c}.$$

In order to investigate the reliability of the theoretical model varying the geometry of the beam, let us rewrite Equation (6) in the following dimensionless form:

(8) $$\begin{array}{ccc} {\bar{m}}_{x}\left(s\right) & = & \beta_{ES}^{-1}\{1-\frac{\beta_{ES}^{-2}}{4E_{\mathrm{MR}}}\{\beta_{C}^{2}\left\{\frac{\nu_{\mathrm{MR}}}{3}a\left(6\nu_{\mathrm{MR}}+3\right)+b\left[4\nu_{\mathrm{MR}}\left(5-2\nu_{\mathrm{MR}}\right)+6\right]+c\left[2\nu_{\mathrm{MR}}\left(7-6\nu_{\mathrm{MR}}\right)+3\right]\right\}+\\ & & \frac{4\nu_{\mathrm{MR}}^{3}\left(8b+23c\right)-\nu_{\mathrm{MR}}^{2}\left(7a+62b+79c\right)-9\nu_{\mathrm{MR}}\left(a+2b+c\right)-6\left(a+2b+c\right)}{5}\}\}+O\left(\beta_{ES}^{-5}\right),\\ & = & \beta_{ES}^{-1}\left[1+\sum_{n=1}^{N}\beta_{ES}^{-2n}\left(\rho_{n0}+\rho_{n}\beta_{C}^{2}\right)\right]+O\left(\beta_{ES}^{-(2N+3)}\right), \end{array}$$

in which ${\bar{m}}_{x}\left(s\right)=12m_{x}\left(s\right)/E_{\mathrm{MR}}BH^{2}$ denotes the dimensionless bending moment, whilst $\beta_{ES}=R_{0}/H$ is the Eulerian slenderness which, together with the compactness index $\beta_{C}=B/H$, governs the problem. Parameters $\rho_{n0}$ and $\rho_{n}$ involves only the constitutive parameters. The form of such parameters for N=2 turns out to be

$$\begin{array}{ccc} \rho_{10} & = & \frac{4\left(8b+23c\right)\nu_{\mathrm{MR}}^{3}-\left(7a+62b+79c\right)\nu_{\mathrm{MR}}^{2}-9\left(a+2b+c\right)\nu_{\mathrm{MR}}-6\left(a+2b+c\right)}{5},\\ \rho_{1} & = & \frac{\nu_{\mathrm{MR}}\left\{\left(6\nu_{\mathrm{MR}}+3\right)a+\left[4\nu_{\mathrm{MR}}\left(5-2\nu_{\mathrm{MR}}\right)+6\right]b+\left[2\nu_{\mathrm{MR}}\left(7-6\nu_{\mathrm{MR}}\right)+3\right]c\right\}}{3},\\ \rho_{20} & = & \frac{1}{840}[2\left(166b+1441c\right)\nu_{\mathrm{MR}}^{5}-\left(31a+1382b+4891c\right)\nu_{\mathrm{MR}}^{4}-135\left(a+2b+c\right)\nu_{\mathrm{MR}}^{3}-\\ & & 390\left(a+2b+c\right)\nu_{\mathrm{MR}}^{2}-450\left(a+2b+c\right)\nu_{\mathrm{MR}}-180\left(a+2b+c\right)],\\ \rho_{2} & = & \frac{\nu_{\mathrm{MR}}}{240}[-8\left(10b+39c\right)\nu_{\mathrm{MR}}^{4}+2\left(15a+190b+343c\right)\nu_{\mathrm{MR}}^{3}+\\ & & 75\left(a+2b+c\right)\nu_{\mathrm{MR}}^{2}+120\left(a+2b+c\right)\nu_{\mathrm{MR}}+60\left(a+2b+c\right)]. \end{array}$$

Relation (8) represents the series expansion of Equation (3.1) of [32] w.r.t. the Eulerian slenderness, thus highlighting the relevance of such a parameter. The Equation (8) truncated at the leading order term, namely $\beta_{ES}^{-1}$, can be interpreted as the Elastica extended to the context of finite elasticity.

It is worth noticing that, in the Lamb theory for shells [28], that Author observed that *“for sufficiently small curvatures, i.e., so long as R is large compared to $B^{2}/H$ the shell profile is close to a paraboloid”*. He found that this situation is encountered for low values of the dimensionless parameter $\sqrt[{4}]{3/4(1-\nu_{MR})}B/\sqrt{R_{0}H}$, being $\nu_{MR}$ the Poisson ratio. As this parameter increases, nonuniform curvature occurs in the cross sections, especially in the neighboring of the corners. Later, based on the Lamb work, Searle [27] experimentally observed that for a high value of the so-called Searle parameter β (it is $\beta^{2}=B^{2}/R_{0}H$), the variation of the deformation field in the transverse cross sections significantly increases. In particular, for high values of β the profile of the cross sections of a bent plate is characterized by an almost flat region in the inner part of the cross section and low values of the anticlastic radius close to the corners. This effect gives rise to the “curl effect”. The condition β=20 was experimentally inferred by Searle in [27] as a threshold value to distinguish among bodies with compact cross sections (beams) and plates.

Based on the above positions, a straightforward relation between the Searle parameter and the Eulerian slenderness can be established by means of the compactness index $\beta_{C}$ according to $\beta^{2}=\beta_{C}^{2}/\beta_{ES}$. As shown in the following, the reliability of the theoretical model to properly describe the mechanical behavior of beams under large bending depends on both the parameters $\beta_{ES}$ and $\beta_{C}$.

### 2.3. The Numerical Procedure

The implementation of the theoretical procedure consists of two main steps: The determination of the deformed axis of the beam by means of Equation (8), which is solved iteratively, and then the assessment of the deformed configuration of the beam axis. This allows the complete description of the 3D kinematics of the bent beam, accounting for the deformation of the transverse cross sections.

It is remarked that, according to [24], in the present work, $R_{0}$ is the radius of curvature of the longitudinal neutral fibre X=0,Y=0 (therefore $R=R_{0}$ and QN=0).

The theoretical model is here implemented for two study cases: A cantilever beam subjected to constant (first study case) and variable (second study case) bending moment. Let us denote with *n* the number of discretizations of the beam axis, and with $s_{i}^{\left(j\right)}$ the *i*th node at the iteration *j*th. Each element (the elements are here assumed equispaced for simplicity, i.e., $\Delta s=L/n=∥s_{i+1}-s_{i}∥$) preserves its original length during deformation, as sketched in Figure 2.

The iteration number corresponding to the achievement of the convergence criterion is denoted by *N*.

Therefore, starting form a prescribed guess solution (tipically, the solution provided by the linearized theory), the radius of curvature $R_{0}$ of the longitudinal axis of the beam is known at a given node $s_{i}^{\left(j\right)}$ through relation (6). Once the radius of curvature is known, the curvature $\chi_{0}\left(s\right)=1/R_{0}\left(s\right)$, provides the rotation θ(s) at each node as

(9) $$\theta \left(s_{i}^{\left(j\right)}\right)=\theta \left(s_{1}^{\left(j\right)}\right)+\frac{\Delta s}{2}\sum_{k=2}^{i}\left[\chi \left(s_{k}^{\left(j\right)}\right)+\chi \left(s_{k-1}^{\left(j\right)}\right)\right],$$

and then the displacement field follows according to:

(10) $$v(s_{i}^{\left(j\right)})=v\left(s_{1}^{\left(j\right)}\right)-\frac{\Delta s}{2}\sum_{k=2}^{i}\left[\sin\theta_{k-1}^{\left(j\right)}+\sin\theta_{k}^{\left(j\right)}\right],$$

(11) $$w(s_{i}^{\left(j\right)})=w\left(s_{1}^{\left(j\right)}\right)-\frac{\Delta s}{2}\sum_{k=2}^{i}\left[2-\cos\theta_{k-1}^{\left(j\right)}-\cos\theta_{k}^{\left(j\right)}\right].$$

Summing up, the present approach is based on the 1D solution of the governing Equation (8), followed by the determination of the displacement field of the transverse cross sections. In detail, the numerical procedure used in Section 3 consists of the following steps:

- starting from a trial solution corresponding to the linearized theory, the bending moment $m_{x}\left(s_{i}^{\left(1\right)}\right)$ is obtained at the first iteration;
- for each node, Equation (6) is solved in the unknown radius of curvature $R_{0}\left(s_{i}^{\left(1\right)}\right)$;
- then, the rotation and displacements fields are assessed from (9)–(11) for each node, providing the Eulerian coordinates of the beam axis, i.e., $\left[z_{i}^{\left(2\right)},y_{i}^{\left(2\right)}\right]=\left[Z_{i}+w\left(s_{i}^{\left(1\right)}\right),\;Y_{i}+v\left(s_{i}^{\left(1\right)}\right)\right]$;
- two convergence criteria have been adopted: The relative error between two subsequent iterations in terms of displacement norm and the potential energy;
- in case of not convergent results, the deformed configuration (not convergent) is used as guest solution for assessing a further bending moment distribution, $m_{x}\left(s_{i}^{\left(2\right)}\right)$. Therefore, the iterative procedure restarts from the second issue since both the convergence criteria are reached.

Given the elastic potential energy Π at iteration *j*-th defined as

$$\Pi^{\left(j\right)}\left(s^{\left(j\right)},\theta^{\left(j\right)}\right)=\frac{\Delta s}{2}\sum_{i=1}^{n+1}\chi \left(s_{i}^{\left(j\right)}\right)\;m\left(s_{i}^{\left(j\right)}\right)-\mu \left\{\begin{array}{cc} M_{0}\;\theta \left(s_{n+1}^{\left(j\right)}\right), & \mathrm{study}\;\mathrm{case}\;1\\ F_{0}\;w\left(s_{n+1}^{\left(j\right)}\right), & \mathrm{study}\;\mathrm{case}\;2, \end{array}\right.$$

the relative errors between subsequent iterations in terms of energy and the displacement norms are defined as

$$\epsilon_{\Pi^{\left(j\right)}}=\frac{\Pi^{\left(j\right)}-\Pi^{\left(j-1\right)}}{\Pi^{\left(j\right)}},\;\epsilon_{s^{\left(j\right)}}=\frac{∥s^{\left(j\right)}∥-∥s^{\left(j-1\right)}∥}{∥s^{\left(j\right)}∥},$$

the solution at the iteration *N*th is convergent if the following criterion holds true:

(12) $$\max\left\{\epsilon_{\Pi^{\left(N\right)}},\;\epsilon_{∥s^{\left(N\right)}∥}\right\}<10^{-8}.$$

## 3. Study Cases

**Figure 3:**
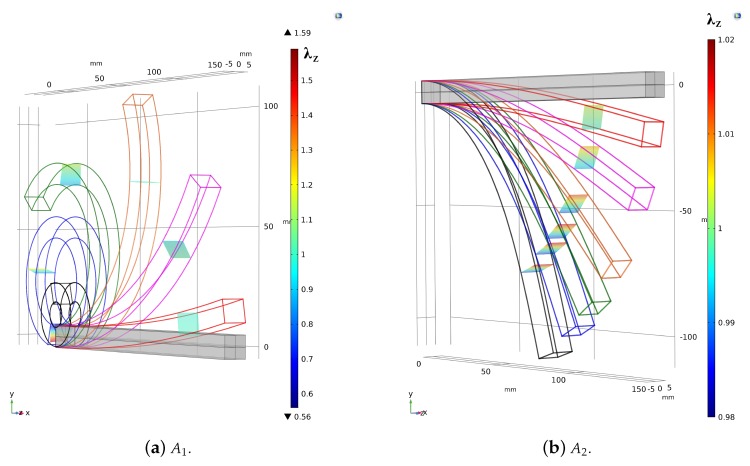
Sketch of the study cases: Reference and deformed configurations provided by the FE solution.

Two study cases are considered here: A clamped beam subjected to a bending couple (case $A_{1}$ sketched in Figure 3a) and a clamped beam subjected to a dead shear force (case $A_{2}$ shown in Figure 3b) acting at the free end.

For each application, three subcases related to three different sizes of the cross section have been considered according to Table 1. Note that both the thickness and length have been fixed, varying the width of the cross sections only.

In the following, notation $A_{2}\left(c\right)$ stands for study case $A_{2}$ with the cross section geometries corresponding to the subcase (c) and so on.

Subcase (a) represents a slender beam with square compact cross section, which properly fulfills the theoretical hypotheses. Subcase (b) represents an intermediate situation of a beam weakly slender, $\beta_{LS}<10$, with a flat cross section ($\beta_{C}=2$). To emphasize the effect induced by loss of slenderness, the subcase (c) resembling a plate ($\beta_{LS}=3$ and $\beta_{C}=5$) is considered also.

The following MR constitutive parameters have been adopted: a=1,b=0.05 and c=2.256 [MPa] according to [32].

The external loads, $M_{0}$ and $F_{0}$ for cases $A_{1}$ and $A_{2}$ respectively, incremented by the load multiplier μ, are such to induce for μ=1 a deflection at the free end of L/100 according to the linearized theory.

For both the study cases, the theoretical model has been implemented by subdividing the longitudinal axis into 100 equispaced elements. The FE simulations involve high computational effort owing to the nonlinear nature of the problem. However, the FE solution provides higher accuracy in terms of equilibrium fulfillment w.r.t. the theoretical formulation, for which the equilibrium has been imposed in the weak form (it is remarked that in the theoretical formulation the equilibrium is satisfied exactly for the sole centroidal fiber).

Conversely to the theoretical model, the FE model accounts for the effect of both the axial or shear load on the deformation field. In other words, the theoretical formulation takes into account only the bending effects induced by the axial or shear forces. In the FE models, the external loads have been simulated indirectly by applying prescribed displacements in order to optimize the achievement of convergence. Therefore, prescribed rotation (case $A_{1}$) or transverse displacement (case $A_{2}$) have been imparted at the final cross section and to its centroid, respectively. As a consequence, a comparison between the theoretical predictions and FE solutions will be performed in terms of stress resultants over the cross sections. The principal stretch and stress distributions within the beam cross section will be discussed in the following sections.

### 3.1. Cantilever Beam Subjected to a Couple at Its Free End (Case $A_{1}$)

#### 3.1.1. Theoretical Solution

**Figure 4:**
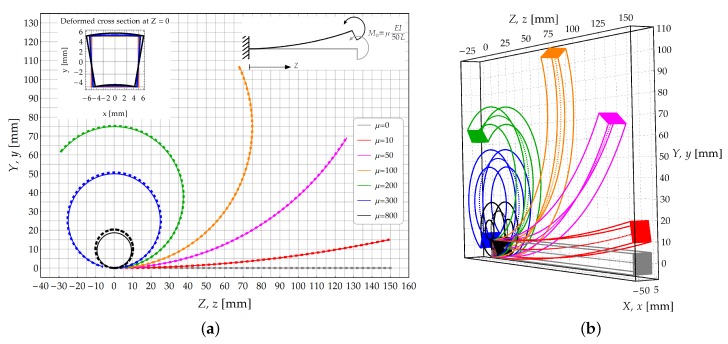
Study case $A_{1}$: Cantilever beam subjected to an external couple acting at its free end. (**a**) Deformed configuration of the longitudinal axis: Theoretical model (continuous lines) and FE model (dashed lines); (**b**) 3D skecth of the deformed configurations obtained by the theoretical solution.

A cantilever beam subjected to an external couple $M_{0}=\mu E_{\mathrm{MR}}I_{X}/50L$ acting at the free end, as sketched in Figure 4, is investigated in the present section. The values assumed by the load multiplier μ have been set according to Figure 4a. Due to the loading condition, the beam is subjected to a constant bending moment $M_{0}$ along its longitudinal axis.

The bending moment distribution w.r.t. the beam axis is obtained as $m_{x}\left(s_{i}\right)=M_{0}$, then the radius of curvature and rotation of the longitudinal axis are achieved by using relations (6)–(9). Finally, the deformed configuration is determined by using Equations (10) and (11) (see Figure 4b).

In order to investigate the effects of bending inside the cross section, stretches and stresses have been computed using Equations (2)–(5).

#### 3.1.2. The FE Model Solution

The FE simulations have been carried out by using COMSOL Multiphysics^®^ v 5.5 software. The non-linear structural mechanics modules of the FE code allows simulating hyperelastic materials. The parametric approach of the FE code makes it possible to define the elastic strain energy function also for compressible materials. Therefore, the MR store energy function (1) has been directly implemented as a function of the principal invariants of the Cauchy-Green strain tensors.

Three FE models (see Figure 3) in agreement with the geometries listed in Table 1, have been modelled by using 4-nodes tetrahedron elements. In this way, each subcase is characterized by 131,282, 211,251 and 308,667 finite brick elements. The compressible MR law (1) is adopted in the FE simulation to compute the (second) Piola Kirchhoff stress tensor $T_{R}^{2nd}=\partial \omega_{MR}/\partial E$ with E=(C−I)/2, being $C=F^{T}F$ the right Cauchy-Green strain tensor and I the identity tensor.

Various tests about the optimal FE mesh have been required in order to obtain displacements at the centroid of the end cross section less than 1% w.r.t. more refined (but more time consuming) meshes.

The clamped end has been reproduced by restraining the out of plane displacement component for each node, namely w(X,Y,0)=0. In addition, the centroidal node at the clamped end has been fully restrained, i.e., s(0,0,0)=0. At the free end, the external couple has been reproduced by imposing a prescribed rotation along the *X* axis. Then, the problem has been handled by increasing step-by-step the prescribed rotation and evaluating a posteriori the corresponding bending moment resultant over the cross section.

#### 3.1.3. Results and Comparison

The deformed configurations of the longitudinal axis are plotted in Figure 4a for different values of the of load multiplier μ. The growth of load multiplier μ is such to induce the beam rolling up on itself, with the simultaneous increase of the beam curvatures.

A first comparison between theoretical predictions and FE results is performed in terms of the bending moment at Z=L. In the FE simulations, the bending moment has been assessed by integrating the elementary moments due to the Cauchy stress $T_{3}$ w.r.t. the *x* axis over the deformed cross section.

As reported in Table 2, the relative error of the FE results w.r.t. the theoretical predictions, denoted as $\epsilon_{r,M_{0}\left(L\right)}$, assumes lower values in correspondence of moderate loads. Indeed, it changes in sign as the load multiplier increases starting from μ=10.

Both the Eulerian slenderness and the compactness of the cross section, represented here through the parameters $\beta_{ES}$ and $\beta_{C}$, affect the reliability of the theoretical model. To highlight this aspect, the values assumed by the Searle parameter $\beta^{2}$ have been listed in Table 2 for each subcase and load increment. In addition, in the same table the values of the fifth-order term in Equation (8), namely $\rho_{20}+\rho_{2}\beta_{C}^{2}$, have been reported also. Since the calculations have been carried out by truncating the moment-curvature relationship (8) to the third-order term, the evaluation of term $\rho_{20}+\rho_{2}\beta_{C}^{2}$ allows assessing the approximation of the performed analysis.

The Searle parameters, here written as the product of the Eulerian slenderness (which varies with the load multiplier) and the compactness of the cross sections (which is independent of the load multiplier), increases as the load multiplier increases due to the decrease of $\beta_{ES}$.

Note that the growth of the Eulerian slenderness decreases the relevance of the high order terms in relation (6). In particular, terms $\rho_{20}+\rho_{2}\beta_{C}^{2}$ listed in Table 2 put in light the relevance of the higher-order terms w.r.t. the first one in the moment-curvature expression. The comparison between theoretical predictions and FE results allows assessing the reliability of the theoretical formulation varying the parameters $\beta_{ES}$ and $\beta_{C}$.

**Figure 5:**
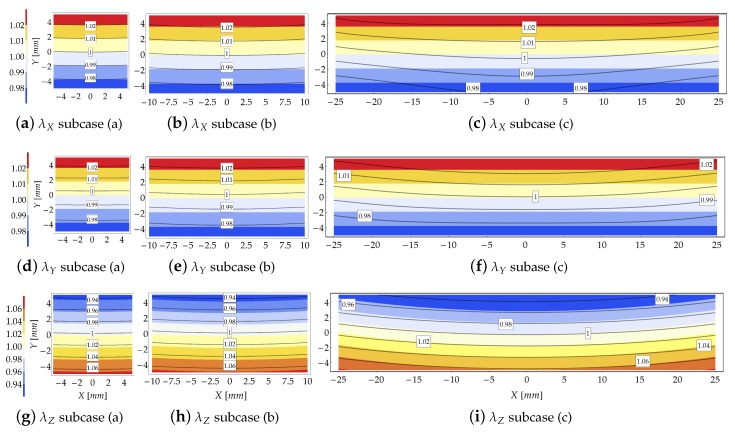
Study case $A_{1}$ at load increment 3 (μ=100): Comparison in terms of stretches provided by the theoretical model (contour plot) and the FE solution (solid isolines with boxed value).

Results provided by the theoretical model and the FE simulations, in terms of principal stretch distributions inside the cross section at Z=0 for μ=100, are shown in Figure 5. The contour plots represent the theoretical results whereas the FE solutions are represented with solid isolines with boxed values. The same contour range has been used for representing the principal stretches.

Moving from compact to flat cross sections, i.e., as $\beta_{C}$ increases (from left to right in Figure 5), the gap between the FE and theoretical results increases. The FE results indicate that as $\beta_{C}$ increases the principal stretches variability w.r.t. the *X* axis (namely, along the cross section width) takes relevance. On the other hand, the comparison reported in Figure 5 highlights the reliability of the theoretical model to predict the kinematics of the bent beam since, in that case, the Searle parameter assumes low values, being $\beta^{2}<0.5$.

For small values of the Searle parameter, the theoretical and FE solutions in terms of transverse stretch $\lambda_{X}$ are almost indistinguishable. In general, the theoretical solution is more reliable in the region close to the core of the cross section, where the theoretical formulation exactly fulfills the equilibrium condition, see Figure 5a,b,d,e,g,h. The gap between the theoretical and FE results slightly increases for the transverse principal stretch $\lambda_{Y}$ w.r.t. stretch $\lambda_{X}$.

**Figure 6:**
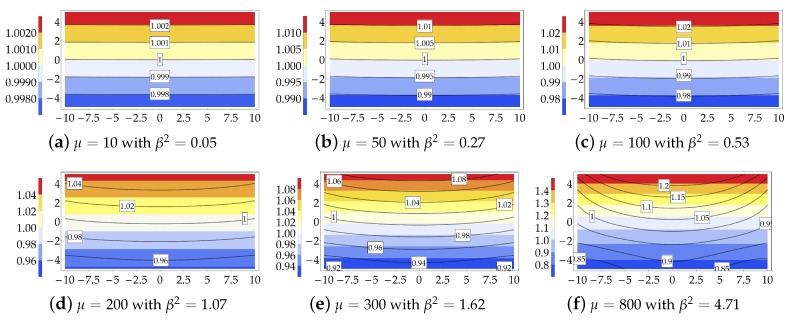
Study case $A_{1}\left(b\right)$: Transversal principal stretch $\lambda_{X}$. Theoretical model (contour plot) and FE simulation (solid isolines with boxed value) for each value of the load multiplier and related values of the Searle parameter.

Conversely, rough theoretical predictions in terms of stretch occur for subcase $A_{1}\left(c\right)$ because of the relevant value assumed by the Searle parameter ($\beta^{2}=3.49$). In order to highlight the effect induced by loss of Eulerian slenderness on the reliability of the theoretical model, Figure 6 shows the increase of the gap between the principal stretches $\lambda_{X}$ provided by the theoretical formulation and the FE simulations for subcase $A_{1}\left(b\right)$.

Figure 6 clearly shows that around the third and fourth load increment a significant gap between the analytical and numerical solutions is encountered.

Another source of mismatch between the theoretical predictions and the FE solutions lies in the approximated nature of the moment-curvature relation (6). It must be remarked that the higher-order terms in the governing equation assume values comparable or greater than the leading order term. Such aspect will be investigated indetail in a forthcoming work.

The distribution of the principal stretch $\lambda_{Z}$ within the cross section is displayed in Figure 5g–i. As expected, the isolines are almost equispaced in agreement with the Euler–Bernoulli beam theory, as confirmed also by the FE results. However, as the slenderness decreases (from left to right in Figure 5), the gap between analytical and numerical isolines increases. Note also that the longitudinal neutral fiber for which $\lambda_{Z}=1$ is not horizontal, but it resembles an arc of circumference.

**Figure 7:**
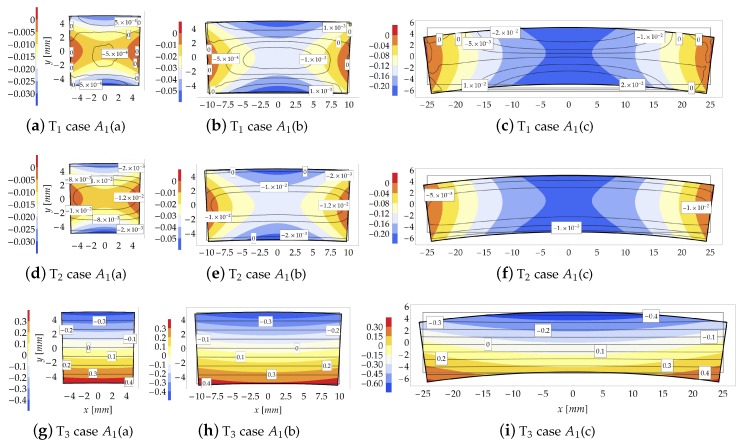
Study case $A_{1}\left(b\right)$ at load increment 3 (μ=100): Stresses comparison [MPa] between the theoretical model (contour plot) and FE model (isolines with boxed value).

The distribution of the Eulerian stresses inside the cross sections is shown in Figure 7 for μ=100. Differently from Figure 5, for sake of graphical representation each contour plot of Figure 7 is provided with a proper contour legend owing to the wide range assumed by stresses.

As the compactness index $\beta_{C}$ increases (from case $A_{1}\left(a\right)$ to case $A_{1}\left(c\right)$), the FE model highlights a sensible change in the distribution of the internal stresses due to “plate effects”, neglected in the theoretical formulation. The FE results indicate that, for $\beta_{C}<0.5$, the transverse principal stresses $T_{1}$ and $T_{2}$, assume values two or three orders lower than that of the principal stress $T_{3}$, Figure 7a,b,d,e. Note also that, for case $A_{1}\left(c\right)$, the transverse stress $T_{1}$ is, in average, higher than that for cases $A_{1}\left(a\right)$ and $A_{1}\left(b\right)$. However, despite the discordance between theoretical and numerical predictions, it is shown that the theoretical model reproduces a main stress $T_{3}$ of the same order of those provided by FE simulations.

### 3.2. Cantilever Subjected to a Shear Force Acting at Its Free end (Case $A_{2}$)

#### 3.2.1. Theoretical Solution

**Figure 8:**
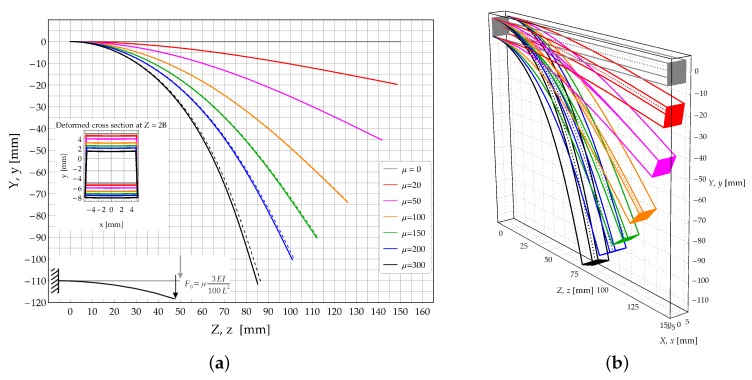
Study case $A_{2}$: Cantilever beam subjected to a transverse dead load acting at its free end. (**a**) Deformed configuration of the longitudinal axis: Theoretical model (continuous lines) and FE model (dashed lines); (**b**) 3D skecth of the deformed configurations obtained by the theoretical solution.

The study case $A_{2}$, related to a cantilever subjected to a transverse shear force acting at its free end is investigated in the present Section. A dead shear load $F_{0}=3EI\mu \;/100L^{2}$ is applied and incremented according to the load multipliers reported in Figure 8a. In that figure the theoretical model (solid lines) and FE solutions (dashed lines) are compared in terms of deformed configurations (see Figure 8b).

Conversely to case $A_{1}$, here the bending moment varies along the longitudinal axis of the beam and it depends on the deformed configuration. Furthermore, in the deformed configuration the shearing dead load produces also axial stresses, see Figure 8a. It is remarked that the theoretical model neglects the effects induced by shear and axial loads on the strain field.

As reported above, the theoretical model is implemented starting with the guess solution corresponding to the solution provided by the linearized elasticity. Once the deformed configuration is known, the bending moment follows from the equilibrium. Then, the obtained bending moment at each node is plugged into relation (6), whose solution provides the longitudinal radius of curvature and, in turn, the rotation and displacements fields according to Equations (9)–(11). At this point, the algorithm restarts until the obtained solution converges according to the criterion given in Equation (12).

As expected, the convergence rate decreases as the load multiplier increases, as confirmed by the iteration number required to reach the convergence, namely N=13,20,40,55,84 and 205, for each load increment, respectively, and it is not significantly affected by the beam geometry.

#### 3.2.2. The FE Solution

The deformed configurations provided by FE simulations are shown in Figure 3b. The geometries listed in Table 1 have been simulated by using 4-nodes tetrahedron elements. Such subcases are characterized by 130569, 212161 and 284231 number of FEs.

Conversely to the study case $A_{1}$, the cross section at Z=0 has been fully restrained (namely s(X,Y,0)=0) to mitigate the noise induced by a single concentrated reactive force at the clamped end. Moreover, the comparison between the theoretical and FE predictions has been performed at Z=2B (the value of 2B has been taken here as an extinction length equals to 2max{B,H}.).

Like the previous study case, the external load is simulated by applying a displacement. In particular, the prescribed displacement values v(0,0,L) corresponding to the load increments considered in the theoretical model are listed in Table 3.

As already observed, the FE models account for the deformations induced by both the axial and shear loads also. In order to quantify such contributes, the following two dimensionless measures of deformation can be introduced

(13) $$\Delta L=\frac{1}{L}\int_{0}^{L}\lambda_{Z}\left(0,\;0,\;Z\right)\;dZ-1,$$

(14) $$\Delta \gamma =\frac{2}{BHL}\int_{B}\sqrt{|C_{YZ}\left(X,\;Y,\;Z\right)|}\;dV.$$

**Figure 9:**
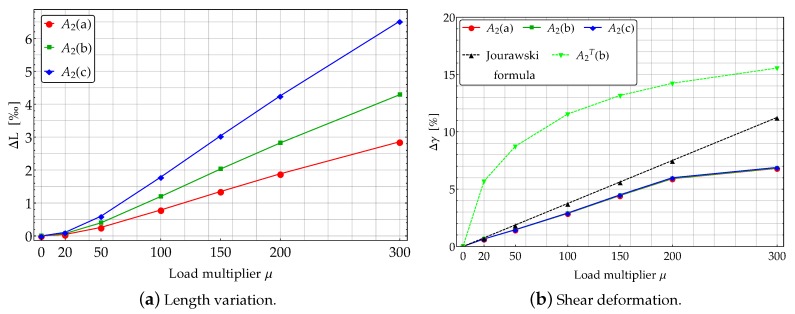
Effects of axial and shear deformations about study case $A_{2}$.

Such quantities, representative of the deformation induced by the axial and shear stresses, have been been reported in Figure 9 varying the load multiplier.

The axial elongation ΔL (13) highlights the length variation associated whit the axial component of the external force. Such a variation turns out to be very small, also for high values of the load multiplier, as shown in Figure 9a. This contribution exhibits a different nonlinear growth with the load multiplier varying the subcases. It follows that the geometry of the beams significantly influences the value of ΔL for $\beta_{C}>1$.

The (global) measure of the shear deformation (14) can be interpreted, according to the linearized elasticity, as average engineering shear strain in the YZ-planes and it quantifies the sliding occurring in the planes of the cross sections. The amount of shear Δγ contributes negligibly to the rotation θ of the cross section. Based on Figure 9b, it could seem that Δγ be much more relevant than ΔL. Nonetheless, the maximum value of Δγ turns out to be only 7% of the final angle of rotation of the cross section due to bending, which is $\theta \left(L\right)=-73^{{}^{\circ}}$.

In Figure 9b $A_{2}^{T}\left(b\right)$ denotes a further subcase for which it has been assumed H=20 mm and B=10 mm. The average contribution of the shear strain seems to be almost independent of the geometry of the beam cross section, as predicted by linearized elasticity. Indeed, according to the well known Jourawski formula [33] in the framework of linearized elasticity, one has

(15) $$\tau_{YZ}\left(Y,L\right)=\frac{F_{0}S_{X}\left(Y\right)}{I_{X}B},$$

in which $\tau_{YZ}$ is the shear stress, $F_{0}$ the external shear force acting at a given beam cross section and $S_{X}\left(Y\right)$ is the first order moment w.r.t. the *X* axis of the part of the cross section overlaying the fibre at distance *Y* form the *X* axis. Therefore, by assuming $C_{YZ}≅\gamma_{YZ}$ it follows that $C_{YZ}≅\tau_{YZ}/G$, with G=E/2(1+ν). Considering the values of the shear load $F_{0}$ reported in Figure 8a and applying the Jourawski formula one finds

(16) $$\begin{array}{ccc} \gamma_{YZ} & = & \mu \frac{3(\nu +1)}{400}{\left(\frac{H}{L}\right)}^{2}=k\mu . \end{array}$$

Therefore, Equation (16) does not depend explicitly by the width *B* of the cross section. Conversely, $\gamma_{YZ}$ significantly depends on the heigth *H* of the cross section, as confirmed by curve of Figure 9b related to case $A_{2}^{T}\left(b\right)$.

#### 3.2.3. Results and Comparison

For each load increment of case $A_{2}$, the deformed configurations of the longitudinal axis of the bent beam are reported in Figure 8a. In that figure, solid lines represent the analytical predictions whilst dashed lines reproduce the FE results. A sketch of the 3D configurations is provided in Figure 8b.

The relative errors of the FE results w.r.t. the theoretical predictions in terms of resultant of the shear stresses are listed in Table 4.

As for the previous case, the relative errors concerning the reactive force provided by the FE solution and the theoretical model keeping fixed the displacement v(0,0,L) start from negative values that increase as the load multiplier increase. This means that the theoretical model provides stiffer response w.r.t. the numerical ones. Indeed the FE solutions accounts for the shear and axial compliance also.

**Figure 10:**
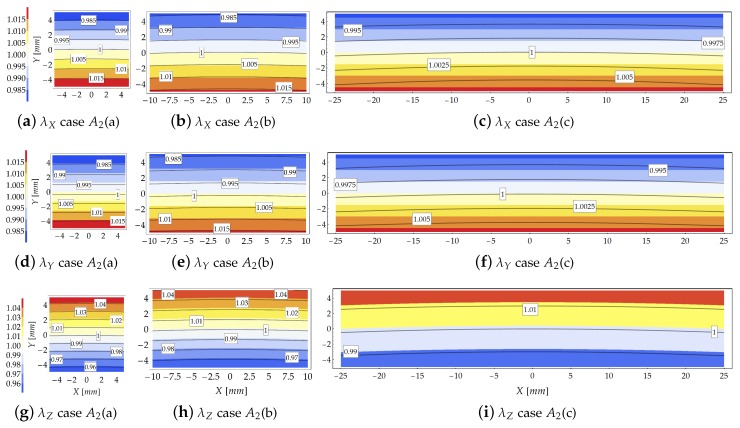
Study case $A_{2}$ at increment 3 (μ=100): Comparison in terms of stretches provided by the theoretical model (contour plot) and the FE solution (solid isolines with boxed value).

The discussion provided in Section 3.1.3 about the reliability of the theoretical model based on the Searle parameter holds also for the case of variable bending moment, as the study case at hand. Indeed, the comparison provided in Figure 10 in terms of stretches displays good agreement between theoretical and numerical predictions for low values of the Searle parameter, see Table 4. In detail, for $\beta^{2}\leq 0.5$ the analytical formulation provides extremely accurate results.

Note that for the study case $A_{2}$, the values of the Eulerian slenderness are lower than those related to case $A_{1}$, as listed in Table 2. It means that, despite of the large values of the displacements, the parameter $\beta_{ES}=H/R_{0}$, is smaller. Conversely to case $A_{1}$, in case $A_{2}$ the bending moment at the clamped side increases slowly as the load multiplier increases owing to the reduction of the external load arm.

For subcases $A_{2}\left(a\right)$ and $A_{2}\left(b\right)$, for which $\beta^{2}<0.5$ (see Table 4), the theoretical and numerical results in terms of stretch distributions within the cross section agree well, as shown in Figure 10. For these cases, as shown in Figure 10a,b,d,e,h,g the theoretical model is able to grasp both the stretches magnitude and their distribution within the cross section. In particular, for case $A_{2}\left(a\right)$ the stretches predicted by the theoretical formulation and those provided by the FE code are almost coincident.

Moving from the subcase (a) to subcase (c) the analytical model reduces its accuracy, leading to an underestimation of the stretches as compared with those furnished by the FE simulations. However, since $\beta^{2}<0.5$ (cases $A_{2}\left(a\right)$ and $A_{2}\left(b\right)$ of Figure 10), the theoretical model preserves its reliability to predict the stretches. For the subcase $A_{2}\left(c\right)$, for which $\beta^{2}=0.81$, the theoretical formulation loses its reliability, as confirmed by Figure 10c,f,i.

**Figure 11:**
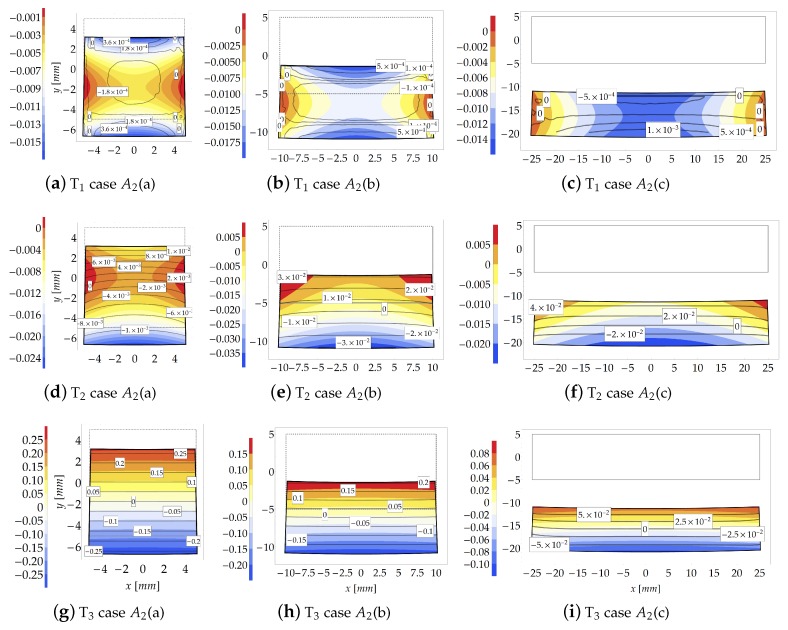
Study case $A_{2}$ at increment 3 (μ=100): Stresses comparison [MPa] between the theoretical model (contour plot) and FE model (isolines with boxed value).

As the compactness index $\beta_{C}$ increases (from left to right in Figure 11), the FE results show that the transverse principal stress $T_{2}$ becomes comparable w.r.t. principal stress $T_{3}$.

As already observed, conversely to the FE solution, the reliability of the theoretical formulation sensibly depends on the geometry of the cross sections. Indeed, for subcases in which $\beta^{2}<0.5$ (see Figure 11c,f), the analytical formulation loses its accuracy to predict stretches and stresses. Moreover, the FE solution indicates that stress $T_{1}$ assumes negligible values w.r.t. the other principal stresses. In particular, the principal stress $T_{1}$ is about one or two orders lower than $T_{2}$, and three orders lower than $T_{3}$ when $\beta_{C}<0.5$.

As expected, the principal stress $T_{3}$ is accurately predicted by the theoretical model when $\beta^{2}<0.5$, as displayed in Figure 11f,h,i.

## 4. Conclusions

The finite bending of a homogeneous hyperelastic beam made of a compressible Mooney–Rivlin material has been investigated here. Two study cases have been analyzed in detail: A cantilever subjected to a prescribed couple and a cantilever subjected to an external shear load acting at its free end. For each study case, the main analytical results provided by the theoretical model [23] have been compared with those obtained by FE simulations.

The governing Equation (6), has been rearranged here in dimensionless form, allow identifying two dimensionless governing parameters here termed Eulerian slenderness $\beta_{ES}=R_{0}/H$ and an index about the compactness of the cross section, i.e., $\beta_{C}=B/H$. Such parameters are involved in the Searle parameter β [27,28] according to the expression $\beta^{2}=\beta_{C}^{2}/\beta_{ES}$.

The comparison between the theoretical and numerical predictions in terms of the deformed configurations of the bent body together with stretch and stress distributions within the cross section, has been provided and discussed. Such comparison allows assessing the accuracy of the theoretical model to predict the mechanical response of beams under large bending. It is shown that the analytical model provides accurate results provided that the following conditions about the governing parameters hold true: $\beta_{C}<0.5$ and $\beta^{2}<0.5$ (namely, $\beta_{ES}<0.125$).

Once the aforementioned threshold values are overcome, the deformed configuration of the cross sections differs from an arc of circumference as hypothesized in the theoretical formulation. This fact is due to relevant transverse deformations according to the theory of elastic plates.

The study cases here considered allows confirming the negligible effects of the shear and axial loads compared with the bending effects. Furthermore, the present study allows validating the simplifying hypotheses involved in the theoretical formulation [23] also for beams subjected to variable bending moment.

The obtained moment-curvature relationship extends the classical Elastica to the framework of finite elasticity. In a forthcoming work, the authors will compare the results provided by Equation (8) with those furnished by the Elastica with the aid of experimental tests on rubber-like materials.

## Figures and Tables

**Table 1:** Dimensionless geometric parameters related the investigated subcases: *H* =10 [mm], $\beta_{C}=B/H$ and $\beta_{LS}=L/\max\left(B,H\right)$.

Subcases	(a)	(b)	(c)
$\beta_{C}$	1	2	5
$\beta_{LS}$	15	7.5	3

**Table 2:** Study case $A_{1}$: Relative error on the bending moment $\epsilon_{r,m_{x}\left(L\right)}$, Searle parameter $\beta^{2}=B^{2}/R_{0}H=\beta_{C}^{2}/\beta_{ES}$ and weight of the fifth-order truncated term in Equation (8).

Load	Subcases								
Mult.	(a)			(b)			(c)		
μ	$\epsilon_{r,m_{x}\left(L\right)}$	$\beta^{2}$	$\rho_{20}+\rho_{2}\beta_{C}^{2}$	$\epsilon_{r,m_{x}\left(L\right)}$	$\beta^{2}$	$\rho_{20}+\rho_{2}\beta_{C}^{2}$	$\epsilon_{r,m_{x}\left(L\right)}$	$\beta^{2}$	$\rho_{20}+\rho_{2}\beta_{C}^{2}$
10	$-3.1\times 10^{-3}$	0.01	$1.6\times 10^{-9}$	$-5.7\times 10^{-3}$	0.05	$-1.2\times 10^{-4}$	$2.8\times 10^{-1}$	0.33	$2.1\times 10^{1}$
50	$-2.4\times 10^{-3}$	0.07	$9.8\times 10^{-7}$	$-5.5\times 10^{-3}$	0.27	$-8.\times 10^{-2}$	$2.7\times 10^{-1}$	1.68	$5.4\times 10^{2}$
100	$2.\times 10^{-4}$	0.13	$1.5\times 10^{-5}$	$-3.9\times 10^{-3}$	0.53	−1.7	$2.6\times 10^{-1}$	3.49	$2.3\times 10^{3}$
200	$1.3\times 10^{-2}$	0.26	$2.3\times 10^{-4}$	$5.6\times 10^{-4}$	1.07	$5.3\times 10^{1}$	$2.3\times 10^{-1}$	19.32	$7.1\times 10^{4}$
300	$2.5\times 10^{-2}$	0.38	$1.\times 10^{-3}$	$6.5\times 10^{-3}$	1.62	$7.4\times 10^{1}$	$2.3\times 10^{-1}$	20.35	$7.9\times 10^{4}$
800	$1.6\times 10^{-1}$	0.87	$2.3\times 10^{-2}$	$5.8\times 10^{-2}$	4.71	$3.1\times 10^{2}$	$3.6\times 10^{-1}$	24.17	$1.1\times 10^{5}$

**Table 3:** Prescribed displacements −v(0,0,L) adopted in the FE simulations.

Load Multiplier μ		20	50	100	150	200	300
**Subcases**	**(a)**	19.643	45.230	73.921	90.333	100.303	111.447
	**(b)**	19.646	45.263	74.039	90.521	100.536	111.737
	**(c)**	19.666	45.494	74.917	91.970	102.423	115.291

**Table 4:** Study case $A_{2}$: Relative error on the shear resultant $\epsilon_{r,F_{0}\left(L\right)}$, Searle parameter $\beta^{2}=B^{2}/R_{0}H=\beta_{C}^{2}/\beta_{ES}$ and weight of the fifth-order truncated term $\rho_{20}+\rho_{2}\beta_{C}^{2}$ in Equation (8).

Load	Subcases								
Mult.	(a)			(b)			(c)		
μ	$\epsilon_{r,F_{0}\left(L\right)}$	$\beta^{2}$	$\rho_{20}+\rho_{2}\beta_{C}^{2}$	$\epsilon_{r,F_{0}\left(L\right)}$	$\beta^{2}$	$\rho_{20}+\rho_{2}\beta_{C}^{2}$	$\epsilon_{r,F_{0}\left(L\right)}$	$\beta^{2}$	$\rho_{20}+\rho_{2}\beta_{C}^{2}$
20	$-4.\times 10^{-3}$	0.02	$1.4\times 10^{-8}$	$-1.3\times 10^{-2}$	0.08	$-5.2\times 10^{-4}$	$-5.\times 10^{-2}$	0.22	9.4
50	$-7.7\times 10^{-3}$	0.05	$4.3\times 10^{-7}$	$-3.2\times 10^{-2}$	0.18	$-1.6\times 10^{-2}$	$-1.4\times 10^{-1}$	0.51	$4.9\times 10^{1}$
100	$-1.6\times 10^{-2}$	0.09	$4.\times 10^{-6}$	$-7.8\times 10^{-2}$	0.31	$-1.5\times 10^{-1}$	$-3.9\times 10^{-1}$	0.81	$1.3\times 10^{2}$
150	$-2.4\times 10^{-2}$	0.12	$1.2\times 10^{-5}$	$-1.3\times 10^{-1}$	0.39	$-4.1\times 10^{-1}$	$-7.2\times 10^{-1}$	0.96	$1.8\times 10^{2}$
200	$-2.9\times 10^{-2}$	0.14	$2.2\times 10^{-5}$	$-1.7\times 10^{-1}$	0.45	$-7.5\times 10^{-1}$	−1.1	1.01	$2.\times 10^{2}$
300	$-3.1\times 10^{-2}$	0.18	$4.7\times 10^{-5}$	$-2.5\times 10^{-1}$	0.52	−1.5	−2.2	1.09	$2.3\times 10^{2}$

## Abbreviations

The following abbreviations are used within the manuscript:

w.r.t.	with respect to
MR	Mooney–Rivlin
